# Phenolic Profile and Antioxidant Activity of Melon (*Cucumis Melo* L.) Seeds from Pakistan

**DOI:** 10.3390/foods5040067

**Published:** 2016-10-17

**Authors:** Alam Zeb

**Affiliations:** Laboratory of Biochemistry, Department of Biotechnology, Faculty of Biological Sciences, University of Malakand, Chakdara 18800, Pakistan; Azebuom@gmail.com; Tel.: +92-945-761-622

**Keywords:** honeydew melon, HPLC-DAD, phenolic compounds, antioxidant activity

## Abstract

Phenolic composition of different extracts of honeydew melon seeds and their antioxidant activity was determined for the first time. Phenolic compounds were identified using a reversed phase high performance liquid chromatography with diode array detection (HPLC-DAD) method. Results showed the identification of five phenolic compounds in water extract namely gallic acid and its derivative, hydroxybenzoic acid and catechin derivatives and caffeic acid. There were nine phenolic compounds identified in methanol–water extract, which are caffeic acid, two vanillic acid derivatives, ellagitanins, quercetin-3-rutinoside, derivatives of syringic acid and ellagic acid. The amounts of gallic acid, caffeic acid and catechin were higher among all phenolic compounds. Total phenolic compounds and radical scavenging activity were higher in water and methanol–water extract than their corresponding methanol extracts. In conclusion, melon seeds are a good source of natural antioxidants with significant biological functions and may serve as food ingredients and as fortifying material for maintaining shelf life.

## 1. Introduction

Honeydew melon (*Cucumis melo* L.) belongs to the family Cucurbitaceae and is a creamy yellowish oval shape fruit. Honeydew melon is a widely used fruit from the Asian to the US market. A fully ripe fruit is highly delicious and thus a highly cultivated plant. The melon is rich in important vitamins, such as riboflavin, thiamine and folic acid [1]. It is also a good source of pro-vitamin A and vitamin C [2]. Researchers have found that the concentrations of soluble solids, sucrose, total sugars, β-carotene, and 5-methyltetrahydrofolic acid varied in different parts of the fruit [3]. The fruit has a very short shelf life and deterioration may occur rapidly [4]. The fruit has a very distinct aroma. The characteristic (*Z*,*Z*)-3,6-nonadien-1-ol and phenylethyl alcohol have imparted fresh and sweet-floral characters, respectively, to honeydew aroma [5]. Kolayli et al. [6] studied the chemical and biochemical properties of standard, hybrid, and grafted melons. The authors found that standard melon has the highest score in terms of taste because of its highest sweetness, high antioxidant activity, total phenolic and ascorbic acid contents.

In the center of the melon fruit, a large amount of seeds is present. The fruit part is consumed, while the seeds are used as waste materials. The seeds have been found to contain 4.5% of moisture, crude fat 25.0%, crude protein 25.0%, crude fiber 23.3%, ash 2.4% and carbohydrate 19.8% [7]. Recently, Petkova and Antova [8] analyzed Bulgarian melon seeds for proximate and lipid composition. These authors showed that melon seeds contain fat (41.6%–44.5%), proteins (34.4%–39.8%), crude fiber (4.5%–8.5%), carbohydrates (8.2%–12.7%), soluble sugars (3.7%–4.2%), and minerals (4.6%–5.1%). The lipid fractions include sterols, phospholipids, and tocopherols. The major fatty acid in lipids was linoleic (51.1%–58.5%), and oleic acid (24.8%–25.6%). The trilinolein (LLL) (31.3%–32.2%), oleoyl dilinolein (OLL) (31.0%–34.0%), and palmitoyl dilinolein (PLL) (14.9%–22.3%) were the major triglycerides. Honeydew melon also contains β-carotene and phytoene [9]. There is, however, a lack of information regarding the phenolic composition of honeydew melon seeds and its antioxidant potential from Pakistan. This paper reported for the first time important phenolic compositions of different seed extracts using high performance liquid chromatography with diode array detection (HPLC-DAD) method and their antioxidant potentials.

## 2. Results and Discussion

### 2.1. Phenolic Composition

**Figure 1:**
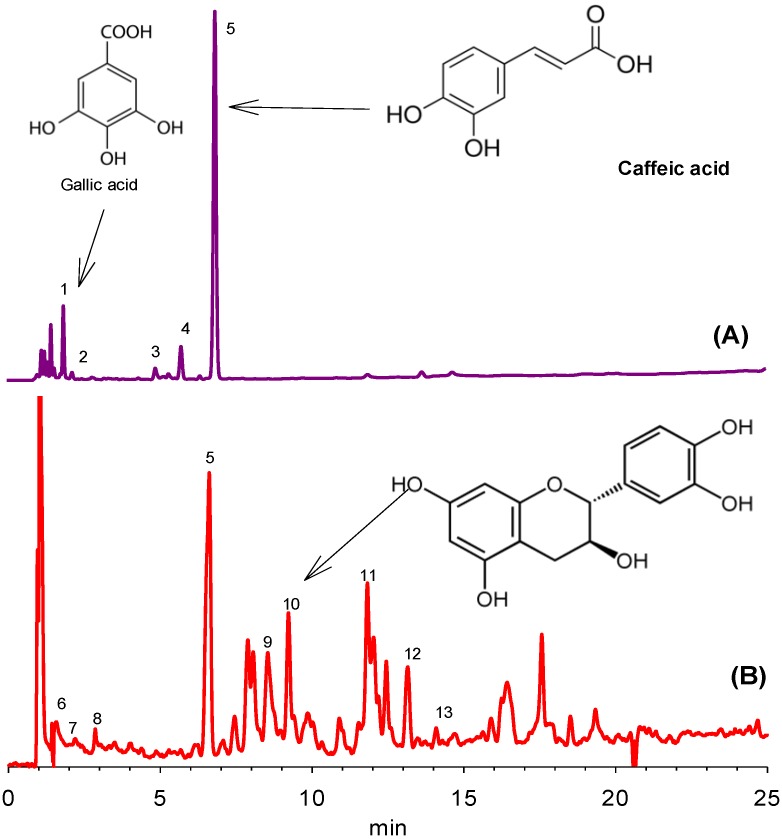
Representative high performance liquid chromatography with diode array detection (HPLC-DAD) chromatograms of the separation of phenolic compounds at 360 nm. (**A**) water extract of melon seeds; and (**B**) methanol–water extract of melon seeds.

**Figure 2:**
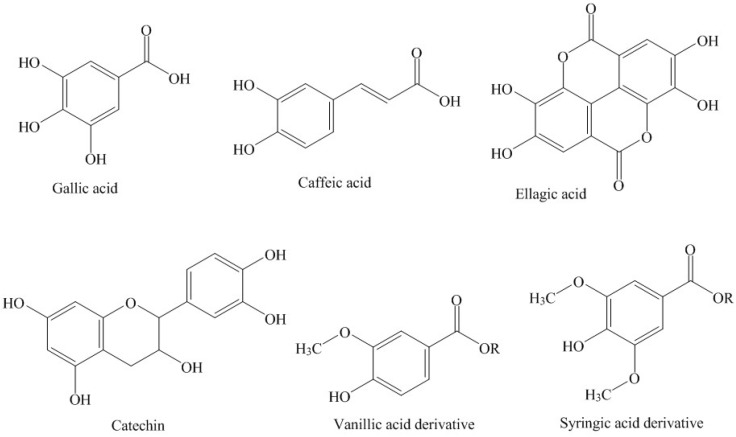
Structure of the major identified phenolic compounds. The R group represents the possible derivative of identified phenolic moiety.

Phenolic compounds were identified using the UV absorption spectra and retention times of the authentic standard compounds or by comparing spectra with those reported in the literature. Only those peaks were selected for quantification where the spectral purity was higher than 96%. The identified compounds were quantified using the standard calibration curves (gallic acid, caffeic acid, quercetin-3-rutinoside, catechin and ellagic acid) and expressed as mg/100 g of the sample. Figure 1 shows the separation of phenolic compounds in water and methanol–water mixer. Each peak number is designated for the specific compound as shown in Table 1. The chemical structures of the major phenolic compounds are shown in Figure 2. Only five compounds were identified in the water fraction (Figure 1A). Compound **1** was eluted at 1.8 min and identified as gallic acid (6.667 mg/100 g). Kolayli et al. [6] also identified gallic acid in different kinds of melon fruits. This shows that gallic acid may be one of the phenolic markers in melon. Compound **2** was tentatively identified as a gallic acid derivative with λmax of 270 nm from the work of Santos et al. [10]. This compound was present in a very small amount (0.982 mg/100 g). Compound **3** eluted at 4.8 min and was found to be hydroxybenzoic acid derivative with λmax of 261 and 278 nm with a concentration of 2.18 mg/100 g. Previous studies [6] showed that hydroxybenzoic acid were only identified in a standard melon variety, and was not detected in grafted and hybrid varieties. These authors reported a high amount of benzoic acid in melon fruits. Similarly, compound **4** was tentatively identified as a catechin derivative with λmax of 280 nm (5.42 mg/100 g). Peak 5 was identified as caffeic acid by comparing the retention time and absorption spectra with authentic standard caffeic acid. The highest amount of caffeic acid was found in the aqueous fraction of honeydew melon seeds (46.3 mg/100 g) among the identified phenolic compounds. Caffeic acid was reported to be present in a very small amount in melon fruits of different varieties [6]. Caffeic acid has several biological roles, of which antioxidant activity is widely known [11]. The HPLC-DAD chromatogram of the methanolic extract of the seeds shows only gallic acid and their derivatives with no other identifiable phenolic compound and was thus excluded to mention in Figure 1. However, the total phenolic compounds present in the methanolic fractions were quantified to be 12.456 mg/100 g, much lower than other fractions.

Figure 1B shows the separation of phenolic compounds in the methanol–water mixed extract. Nine phenolic compounds were identified in mixed extract. A relatively large amount of caffeic acid was found in this extract with concentration of 66.0 mg/100 g. Compounds **6** and **7** were found to be vanillic acid derivatives as identified from the work of Santos et al. [10]. Vanillic acid was previously reported in different kinds of melon fruit by Kolayli et al. [6]. Similarly, compound **8** was tentatively identified as ellagitanin with λmax of 232 and 270 nm (0.433 mg/100 g). Compound **9** was identified as quercetin-3-rutinoside with λmax of 355 and 254 nm and a concentration of 3.91 mg/100 g. This compound was identified by comparing the retention time and absorption maxima of the authentic standard compound. The results of the human studies showed that Quercetin-3-rutinoside is extensively metabolized to 3-hydroxyphenylacetic acid (36%), 3-methoxy-4-hydroxyphenylacetic acid (8%) and 3,4-dihydroxyphenylacetic acid (5%) in humans [12]. The peak 10 was identified as catechin with a concentration of 4.33 mg/100 g, which was found to elute at 9.2 min. Catechin was not reported in different kinds of melon fruits, while a small amount of epicatechin was quantified in a standard variety of melon [6]. Similarly, compound **11** was tentatively identified as a syringic acid derivative with a concentration of 8.65 mg/100 g. Syringic acid was previously identified in the standard melon variety [6], which suggests that this phenolic compound may be metabolized further to form its derivatives in seeds. Compounds **12** and **13** were tentatively identified as ellagic acid derivative (1.24 mg/100 g) and ellagic acid (6.52 mg/100 g). Ellagic acid was identified by comparing the UV absorption spectra and retention time of the standard ellagic acid. Ellagic acid act as a strong antioxidant against human low density lipoproteins (LDL) oxidation [13] and has in vitro anti-proliferative and apoptotic properties. Honeydew melon seeds have a small quantity of ellagic acid and may thus serve as a good source of these natural polyphenolic compounds.

### 2.2. Total Phenolic Contents

**Figure 3:**
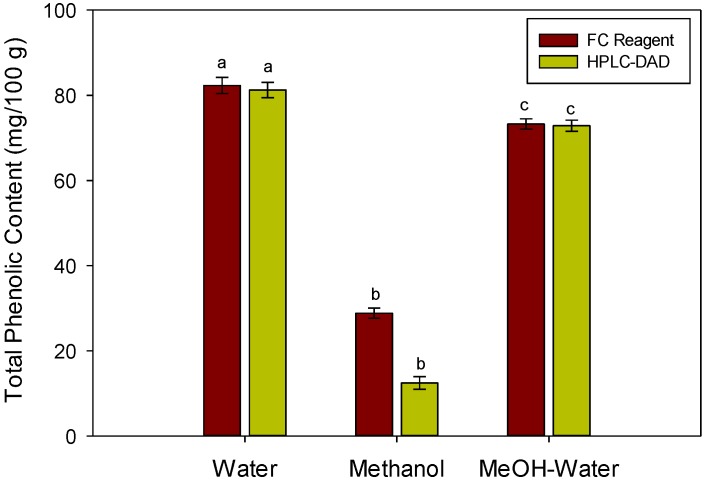
Total phenolic contents (TPC) of the different extracts of melon seeds. Values are mean of triplicate readings. Different letters (a–c) in the same procedure represent significant at *p* < 0.05 (Holm–Sidak method).

A standard gallic acid calibration curve was prepared in the concentration of 5–100 mg/mL. The calibration equation was y = 0.0094x + 0.0086 with *R*^2^ of 0.9976. Figure 3 shows the total phenolic contents (TPC) of the water, methanolic and methanol–water mixer using Folin Ciocalteau (FC) reagent and HPLC. It was observed that the water extract has significantly higher TPC than its corresponding methanolic extract, while the methanol–water mixer has significantly higher TPC than the methanolic extract and lower than the water extract. The results are in agreement with Ismail et al. [14], who determined that the seeds of the melon have the lowest TPC as compared to the methanolic extracts of skin, stem and leaf. There was no significant difference (*p* < 0.05) in the TPC values analyzed with FC reagent and HPLC for water and water–methanol mixture. However, the amount of TPC was significantly lower than the values obtained using FC reagent. This might be due to the lower sensitivity of the detector for the methanolic extract of the melon seeds. The TPC of seeds reported here is significantly higher than the values reported by Chun et al. [15]. This suggests that melon seeds are richer in TPC than their corresponding fruit or plant parts.

### 2.3. Antioxidant Activity

**Figure 4:**
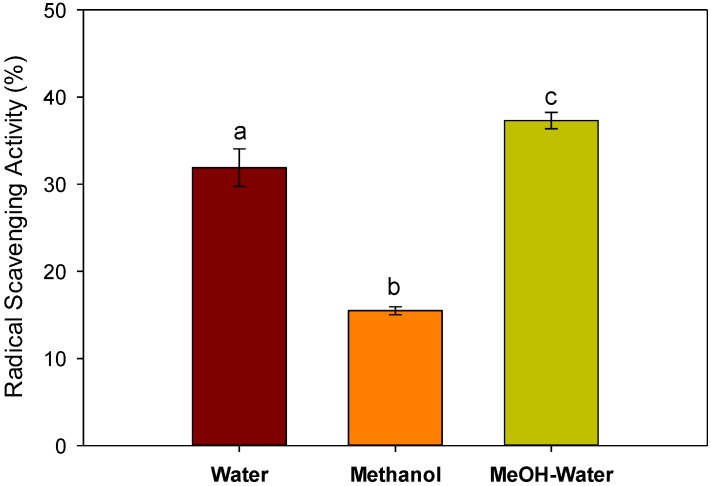
Radical scavenging activity (RSA) of the different extracts of melon seeds. Values are the mean of triplicate readings. Different letters (a–c) represent significant at *p* < 0.01 (Holm–Sidak method).

Antioxidant activity was determined using diphenyl picryl hydrazine (DPPH) radicals’ scavenging potential as shown in Figure 4. It was observed that radical scavenging activity of the methanol–water extract was higher than its corresponding pure water and methanolic extracts. The pure methanolic extract has a lower activity than the water extract. Previous work [15] showed that the radical scavenging activity of the fruit was 17.5%, which is lower than the values reported here for water and methanol–water extract, and is relatively similar to the methanolic extract of seeds. Ismail et al. [14] showed that the methanolic extract of the seeds has the highest DPPH radical scavenging activity than methanolic extracts of leaf, stem, skin and flesh of the melon. The high antioxidant activity of the water and methanol–water extract may be due to the high amounts of caffeic acid present in these extracts. The antioxidant potential of other reported phenolic compounds cannot be ruled out, which may also contribute potentially.

## 3. Materials and Methods

### 3.1. Materials

Fresh melon was collected from the market, and its seeds were removed. Gallic acid, caffeic acid, ellagic acid and catechin were from TCI (TCI, Tokyo, Japan). Quercetin-3-rutinoside and all other chemicals were of HPLC grade and were obtained from Sigma-Aldrich (Steinheim, Germany), or as otherwise mentioned.

### 3.2. Sample Collection *and* Preparation

The seeds were removed from the fruits. The seeds were washed with distilled water and were completely dried. The dried seeds were crushed, grinded and converted into finely divided powder. The samples for analysis were prepared from the powdered materials.

### 3.3. Preparation of Extracts

Extracts were prepared in methanol and water (1:1, *v*/*v*). Briefly, 1 g of powdered sample was mixed with 20 mL of methanol–water mixture. The mixture was placed on a water bath at 30 °C for one hour. Then, the samples were centrifuged at 4000 rpm for 10 min. After that, 2 mL from each sample was filtered using Agilent membrane polytetrafluorethylene (PFTE) (Agilent Technologies, Waldbronn, Germany) with pore size of 0.45 µm and transferred into 2 mL Agilent HPLC vials. Similarly, two more extracts in 100% methanolic and 100% pure deionized water were also prepared using similar procedures. One part of the above extracts were used for the determination of total phenolic contents (TPC) using Folin–Ciocaltaeu Reagent. The results of TPC for both methods of analyses were compared.

### 3.4. HPLC-DAD Analyses of Phenolic Compounds

The phenolic profile of the melon seeds was determined using the Agilent Infinity Better 1260 HPLC system (Agilent, Waldbronn, Germany) having a quaternary pump, degasser, auto-sampler and DAD. The separation of the phenolic compounds was achieved using an Agilent Zorbax Eclipse C18 (406 × 250 mm, 5 µm) column. The binary gradient system consists of solvent A (methanol:acetic acid:deionized water, 10:2:88, *v*/*v*/*v*) and solvent B (methanol: acetic acid: deionized water, 90:2:8, *v*/*v*/*v*). The gradient program used was started from 100% A to 85% A at 5 min, and then 50% A at 20 min, 30% A at 25 min, and 100% B from 30 to 40 min [16]. The elution was achieved at 25 min due to the elution of all compounds present. The DAD was set to 280 nm for analysis of phenolic compounds. The spectra were recorded from 190 to 500 nm. The identification and quantification of phenolic compound was carried out using available standards, retention times, and their UV spectra. In cases where an authentic standard was not available, compounds were identified by comparing UV spectra with reported literature and quantified using the available relevant standard phenolic compounds based on the similar chromatographic response as given earlier [10].

### 3.5. Total Phenolic Contents

Total phenolic contents (TPC) were determined using the Folin–Ciocalteu reagent. The reagent was freshly prepared in the lab. Briefly, a sample (0.5 mL) was mixed with 1.5 mL of FC reagent and reacted in the presence of salt for 1 h. Absorbance of the resultant mixture was recorded at 765 nm. A Gallic acid standard calibration curve was prepared in the concentration range of 5–100 mg. The TPC measured was expressed as mg of GAE/100 g.

### 3.6. Radical Scavenging Activity

Radical scavenging activity (RSA) was measured using DPPH radicals. DPPH solution of 0.1 mM was prepared in methanol. DPPH solution of 1.90 mL was mixed with 10 μL of methanolic extracts of the leaves. The mixture was kept in the dark for 30 min and the absorbance was measured at 515 nm using Pharmaspec 1700 spectrophotometer (Shimadzu, Tokyo, Japan). The percent RSA of the samples were calculated using the equation: %RSA = (Ac − As)/Ac, where Ac is the absorbance of control and as is the absorbance of the test sample.

### 3.7. Data Analyses

All samples were measured in triplicate. Data were analyzed for variation by a one-way analysis of variance (ANOVA) and Holm–Sidak method at α = 0.05 using Graph Pad Prism 5 for Windows version 5.03 (Graph Pad Software Inc., La Jolla, CA, USA).

## 4. Conclusions

In conclusion, different extracts of honeydew melon seeds were analyzed for phenolic composition, total phenolic contents and antioxidant activity. The HPLC-DAD results revealed the identification of five phenolic compounds in water extracts, namely gallic acid and its derivative, hydroxybenzoic acid and catechin derivatives, and caffeic acid. The methanol–water extract has revealed nine phenolic compounds, which include caffeic acids, two vanillic acid derivatives, ellagitanins, Quercetin-3-rutinoside, derivatives of syringic acid and ellagic acid. The amounts of gallic acid, caffeic acid, and catechin were higher among all phenolic compounds. Total phenolic compounds and radical scavenging activity were higher in water extract and methanol–water extract than its corresponding methanol extracts. These results concluded that melon seeds are a good source of natural antioxidants and may serve as food ingredients and fortification for maintaining shelf life.

## Figures and Tables

**Table 1:** Identification and quantification of phenolic compounds in honeydew melon seeds using reversed phase HPLC-DAD. Peaks 1–5 were identified in water extract and peaks 5–13 in water-methanol extract.

Peak	Retention Time (min)	Identity	HPLC-DAD λmax (nm)	Amount (mg/100 g)	Reference
1	1.8	Gallic acid	271	6.667	Standard
2	2.1	Gallic acid derivative	270	0.982	Santos et al. [10]
3	4.8	Hydroxybenzoic acid derivative	261, 278	2.18	Santos et al. [10]
4	5.7	Catechin derivative	280	5.42	Santos et al. [10]
5	6.8	Caffeic acid	298, 323	46.3	Standard
5	6.8	Caffeic acid	298, 323	66.0	Standard
6	1.4	Vanilic acid derivative	227, 258	1.02	Santos et al. [10]
7	1.6	Vanilic acid derivative	226, 259	0.455	Santos et al. [10]
8	2.8	Ellagitanin	232, 270	0.433	Santos et al. [10]
9	8.5	Quercetin-3-rutinoside	254, 355	3.91	Standard
10	9.2	Catechin	280	4.33	Standard
11	11.8	Syringic acid derivative	284	8.65	Santos et al. [10]
12	13.5	Ellagic acid derivative	286, 320	1.24	Santos et al. [10]
13	14.3	Ellagic acid	254, 368	6.52	Standard
		Total Amounts	Water	81.249	
			Mixture	72.858	
			Methanol	12.456	

## Abbreviations

HPLC-DAD: high performance liquid chromatography with diode array detection; TPC: total phenolic contents; RSA: radical scavenging activity; GAE: gallic acid equivalent; DPPH: diphenylpicryl hydrazine.
